# Variations of the trapezius branch of the accessory nerve: an anatomic study

**DOI:** 10.1038/s41598-023-47031-w

**Published:** 2023-12-15

**Authors:** Matthew E. Lin, Celeste Kim, Adam Howard, Niels Kokot

**Affiliations:** 1 Keck School of Medicine of the University of Southern California, Los Angeles, CA USA; 2 Department of Otolaryngology, Emory University School of Medicine, Atlanta, GA USA; 3 Department of Otolaryngology-Head and Neck Surgery, Oregon Health and Science University, Portland, OR USA; 4 Caruso Department of Otolaryngology-Head and Neck Surgery, Keck School of Medicine, University of Southern California, 1540 Alcazar Street, Suite 204Q, Los Angeles, CA 90033 USA

**Keywords:** Surgical oncology, Musculoskeletal system

## Abstract

Although modified radical neck dissections have increased in popularity to reduce morbidity secondary to intraoperative accessory nerve damage, inadvertent injury still often occurs. As this phenomenon is thought to be due to anatomic variation in the trapezius branch of the accessory nerve, it is imperative to better understand the nuances of these anatomic variations to better inform surgical decision-making. A total of 24 accessory nerves were dissected, exposed, and traced in 15 cadavers. Three aspects of the accessory nerve were identified and recorded: the course of the trapezius branch in relation to the sternocleidomastoid, the number of trapezius branches at muscle insertion, and the number of cervical rootlet contributions. Four different anatomic patterns for the trapezius branch were identified, with the most common being where the trapezius branch separates from the main accessory nerve just medial to the sternocleidomastoid and courses deep to the sternocleidomastoid (58.3%). Most (75%) trapezius branches entered the muscle as a single nerve, whereas some (21%) were inserted as two separate nerves. The number of cervical rootlet contributions for each trapezius branch varied from zero to three. Bilateral anatomic variations were also noted. Even when the accessory nerve and its branches are thought to be spared during neck dissection, patients may postoperatively present with different degrees of accessory nerve damage. There may be unrecognized anatomic pathways that the nerve takes that may confer a higher risk of unintentional damage, especially those that have greater exposure within the anterior triangle unprotected by the sternocleidomastoid.

## Introduction

Radical neck dissection (RND) was first detailed in 1906 by Crile^1^. He described the en bloc excision of structures including the accessory nerve (AN), a treatment believed to be necessary for adequate oncologic treatment^2^. In 1961, Nahum et al. noted functional disability of the shoulder on the side of radical neck dissection, as patients presented with marked shoulder droop with limited shoulder abduction postoperatively^3^. This constellation of symptoms, also referred to as accessory nerve dysfunction (AND), was attributed to the partial denervation of the trapezius due to intraoperative dissection of the AN and/or its trapezius branches^3^. Further support for this etiology was provided by a study by Short et al. which they compared the functional outcomes between patients who had undergone neck dissection—one with AN preservation and one without—and found superior functional outcomes when the nerve was spared^4^.

Due to the postoperative morbidity that accompanies AN resection during RND, head and neck surgeons now take a modified or selective surgical approach to neck dissections when possible. One of the goals of modified RND is preservation of the AN; however, the nerve is still often inadvertently injured, with an estimated 70% of neck dissections leading to iatrogenic AND^5^. A significant contributor to this high incidence is the anatomic variation of the accessory nerve and its trapezius branches through the anterior and posterior triangles of the neck as well as the varying number of trapezius branches that ultimately reach the muscle^5–8^. As such, it is important to further analyze and detail these variations to prevent accidental nerve damage and minimize postoperative shoulder morbidity.

There is also significant variation in the number of cervical rootlet contributions to the AN and their role in the varied presentations of AND^2^. Some studies show that the cervical rootlets play an important role in the innervation of the trapezius; with the varying number of contributions, damage to these nerves can lead to different levels of postoperative functional morbidity^7,8^. With the many discrepancies in anatomic AN patterns and the high incidence of AND despite efforts to actively preserve the AN, it is imperative to further analyze and describe these pathways. The primary objective of our study is to anatomically characterize variations of the trapezius branch of the AN relative to the SCM. Secondary objectives include identifying the number of cervical rootlet contributions, the number of trapezius branches at muscle insertion, and the variability between the left and right sides.

## Materials and methods

A total of 24 spinal accessory nerves (n = 24) were dissected in 15 donor bodies. The donor bodies were under the care of the Anatomy Department of the Keck School of Medicine of the University of Southern California. The nerves were exposed in standard fashion as in a neck dissection and traced from the skull base, through the sternocleidomastoid, and to their insertion into the trapezius muscle. Three aspects of the AN were identified and recorded: the course of the trapezius branch in relation to the SCM, the number of trapezius branches at muscle insertion, and the number of cervical rootlet contributions.

### Ethical approval

This study was exempt from review by the University of Southern California Institutional Review Board.

## Results

### Trapezius branch anatomic patterns

**Figure 1:**
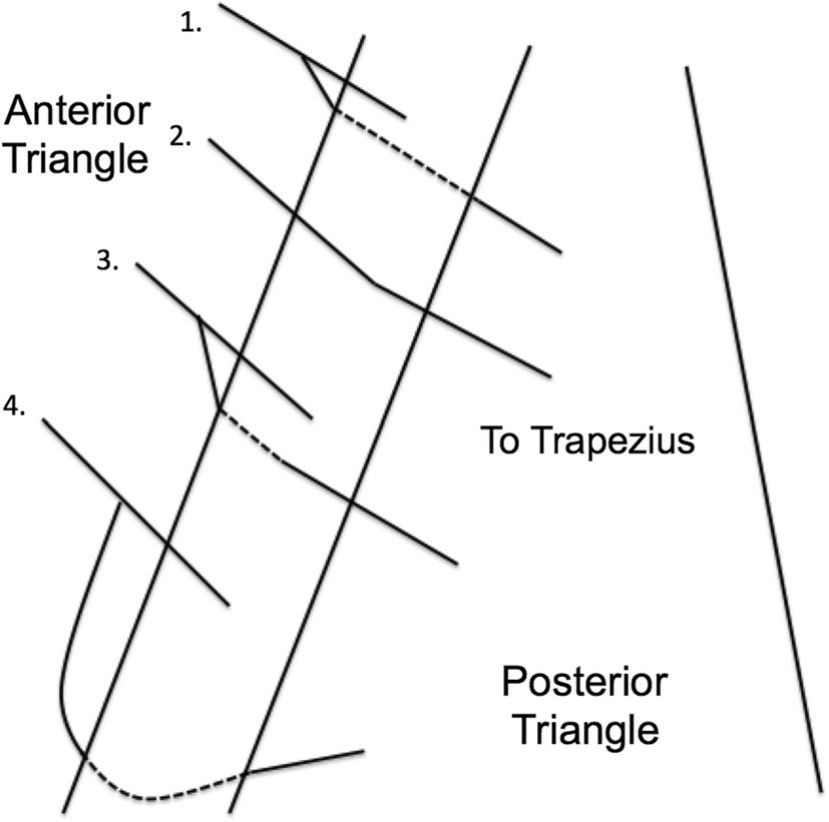
Distribution of trapezius branch variants observed (n = 24): (1) trapezius branch before then deep to SCM; (2) trapezius branch within the SCM; (3) trapezius branch before then through the SCM; (4) trapezius branch running inferiorly then deep to SCM.

Four different anatomic patterns for the trapezius branch of the AN were identified (Fig. 1). A majority (58.3%) of trapezius branches displayed pattern Type 1, where the trapezius branch separates from the main AN just medial to the SCM and courses deep to the SCM. Eight trapezius branches (33.3%) showed pattern Type 2, in which the trapezius branch extends from the AN within the SCM. One branch (4.2%) exhibited pattern Type 3, in which the trapezius branch forms medial to the SCM and pierces through the muscle before continuing to the trapezius. One other branch (4.2%) showed Type 4, where the trapezius branch courses inferiorly along the medial side of the SCM before traveling deep to the SCM.

### Number of trapezius branches at insertion into trapezius muscle

There were also discrepancies in the final number of branches that insert into the muscle. Each trapezius muscle showed either one or two separate nerves, except for one trapezius branch insertion site that could not be commented on. Most (75%) trapezius branches entered the muscle as a single nerve, whereas some (21%) inserted as two separate nerves.

### Number of cervical rootlet contributions

The number of cervical rootlet contributions for each trapezius branch varied from zero to three, with an average of 1 rootlet contribution. Nine (37.5%) trapezius branches had zero cervical contributions, six branches (25.0%) had one contribution, eight branches (33.3%) had two contributions, and one branch (4.2%) had three contributions.

### Anatomic variations bilaterally

Nine bodies had the trapezius branch available for analysis bilaterally, each of which had variations in at least one measured category: trapezius branch patterns, number of branches at trapezius muscle insertion, and/or number of cervical contributions. Three of the nine (33.3%) had bilateral trapezius branches that displayed different pathway patterns, two (22.2%) had varying number of branches inserting into muscle, and seven (77.8%) had different numbers of cervical contributions to the trapezius branches bilaterally.

## Discussion

During radical neck dissection (RND), the tumor and neighboring accessory nerve (AN) are resected^1^. Patients undergoing this procedure postoperatively present with “shoulder syndrome:” a set of symptoms resulting from AND^3,9^. Many authors attribute this condition primarily to trapezius muscle denervation that results from excision of the AN^3,6^. As such, head and neck surgeons often opt for modified or selective neck dissections, during which AN is preserved, to reduce postoperative functional morbidity. This is especially true after one study found sparing the nerve in cases where the AN has not been infiltrated by tumor does not compromise oncologic control^2^.

### Trapezius branch patterns

Iatrogenic damage to the AN often leads to significantly higher morbidity in terms of both pain and function^4^. Although modified radical neck dissection generally offers improved functional outcomes given the preservation of the AN, there are still many patients who are affected by AND postoperatively. Some studies estimate that the AN is unintentionally injured in up to 71% of neck dissections^5^. This high incidence is likely in part due to the unrecognized variations of the AN and its anatomical pathways, such as the branching patterns of the nerves that reach and innervate the trapezius muscle^2,7,8^. As such, identifying these variations is vital for minimizing iatrogenic damage to the AN and subsequent functional morbidity.

Our study identified four distinct branching patterns of the trapezius nerves from the AN main trunk in relation to the SCM (Fig. 1). Most (96%) the trapezius branches were protected by the SCM, as they either traveled posterior to the SCM immediately after separating from the AN main trunk (Type 1 and 3) or branched off within the SCM itself (Type 2). However, one type of trapezius branch pathway initially coursed in a loop anteromedial to the SCM before traveling deep to the muscle (Type 4), a pathway which may make the trapezius branch more vulnerable to inadvertent damage during neck dissection given the greater exposure outside of the SCM in the anterior triangle. As such, this specific course type could help explain the high rates of unintended nerve injury and postoperative AND.

There are limited studies on AN branching patterns; existing ones do not identify the same degree of variation in AN branching patterns in relation to the SCM as our study does, as most others only identify up to three types^6–8^. Furthermore, Type 4 in our study has not yet been recognized elsewhere in existing literature. Lanisnik et al. detailed three variations: trapezius branch separates from the main trunk within the SCM and exits posterior to the SCM, after which it either travels directly to level V or instead travels more medially before going to level V and the trapezius; or, the trapezius branch divides before reaching the SCM and exits posterior to the muscle^7,8^. Alternatively, Shiozaki et al. categorized the branching patterns based on three different degrees of penetration (none, partial, complete) through the SCM, with the partially penetrating type accounting for the largest percentage^6^. A more comprehensive understanding of these anatomic variations can help minimize the amount of unintentional intraoperative nerve damage.

Additionally, our results indicate intra-donor body variability and highlight the relatively unpredictable nature of trapezius branch anatomic patterns. About a third of the donor bodies in our study displayed different branching types on the left and right neck. This degree of bilateral inconsistency emphasizes the fact that anatomic symmetry should not be assumed for the AN and its branches, an assumption which may contribute to intraoperative nerve injury in cases of bilateral neck dissection.

Furthermore, the distribution of the number of branches that ultimately innervate the trapezius muscle is similarly variable. With the exception of one non-observable side, most donor bodies exhibited one trapezius branch (75%), with some showing two (21%) branches, piercing into the muscle. Similarly, other studies have also shown a wide distribution in the number of branches that innervate the trapezius. Kierner et al. found that nine percent utilize one branch, 61% use two branches, and 30% possess three branches^5^. A study by Symes et al. found an even greater degree of variation, with most patients endorsing two or three branches but some showing up to five branches^9^. This high inconsistency of AN anatomic routes and branching patterns of may also help explain the higher rates of inadvertent damage and postoperative AND.

### Cervical contributions

Disparities persist in the existing literature regarding the source of innervation of the trapezius muscle, as some studies suggest cervical nerves contribute greatly to the muscle’s motor innervation^7,10^. As such, patients may present with AND even when the AN and trapezius branches are preserved due to intraoperative severing of cervical contributions during neck dissection^8,10^. Furthermore, different degrees of partial damage to the cervical nerves may help explain the varying postoperative presentations of trapezius denervation^10^. Furthermore, discrepancies also exist in the literature regarding the number of cervical nerves that contribute to the AN and its branches. While Kierner et al. reported two cervical contributions as their most common variant, Symes et al. most one cervical contribution to be the most common^5,9^. In our study, donor bodies most frequently possessed no cervical contributions, with the next most common finding being two contributions. Additionally, from the nine donor bodies eligible for bilateral observation, the greatest amount of intra-donor body variation (78%) was in the number of cervical contributions. These discrepancies in distribution further emphasize the need for additional study of how damage to the cervical contributions affects postoperative functional morbidity.

### Limitations

The use of donor bodies in this study may limit the generalizability of our data, and the lack of clinical correlations limit the applicability of our findings. Furthermore, some of the donor bodies used in the study were previously utilized for other medical education uses, and as such may not have been suitable for bilateral neck dissection at the time of investigation. Future studies should prospectively analyze associations between anatomic patterns and postoperative functional morbidity to evaluate whether specific patterns truly confer higher risks of nerve injury and subsequent AND.

## Conclusions

Even when the AN and its branches are thought to be spared during neck dissection, patients may present with different degrees of AND postoperatively. Our results suggest there may be unrecognized anatomic pathways the nerve takes that may confer a higher risk of unintentional damage, especially among patients with greater exposure within the anterior triangle unprotected by the SCM. Other variations in number of trapezius branches and cervical contributions may also help explain the varying presentations in functional morbidity.

## Data Availability

The datasets used and/or analysed during the current study available from the corresponding author on reasonable request.

## References

[1] Crile G (1987). Landmark article Dec 1, 1906: Excision of cancer of the head and neck. With special reference to the plan of dissection based on one hundred and thirty-two operations. By George Crile. JAMA.

[2] Lloyd S (2007). Accessory nerve: Anatomy and surgical identification. J. Laryngol. Otol..

[3] Nahum AM, Mullally W, Marmor L (1961). A syndrome resulting from radical neck dissection. Arch. Otolaryngol..

[4] Short SO, Kaplan JN, Laramore GE, Cummings CW (1984). Shoulder pain and function after neck dissection with or without preservation of the spinal accessory nerve. Am. J. Surg..

[5] Kierner AC, Zelenka I, Heller S, Burian M (2000). Surgical anatomy of the spinal accessory nerve and the trapezius branches of the cervical plexus. Arch. Surg..

[6] Shiozaki K, Abe S, Agematsu H, Mitarashi S, Sakiyama K, Hashimoto M, Ide Y (2007). Anatomical study of accessory nerve innervation relating to functional neck dissection. J. Oral Maxillofac. Surg..

[7] Lanisnik B, Zargi M, Rodi Z (2014). Identification of three anatomical patterns of the spinal accessory nerve in the neck by neurophysiological mapping. Radiol. Oncol..

[8] Lanišnik B (2017). Different branching patterns of the spinal accessory nerve: Impact on neck dissection technique and postoperative shoulder function. Curr. Opin. Otolaryngol. Head Neck Surg..

[9] Symes A, Ellis H (2005). Variations in the surface anatomy of the spinal accessory nerve in the posterior triangle. Surg. Radiol. Anat..

[10] Pu YM, Tang EY, Yang XD (2008). Trapezius muscle innervation from the spinal accessory nerve and branches of the cervical plexus. Int. J. Oral Maxillofac. Surg..

